# Laser phototherapy induces angiogenesis in the periodontal tissue after delayed tooth replantation in rats

**DOI:** 10.4317/jced.54499

**Published:** 2018-04-01

**Authors:** Felipe-de Souza Matos, Fernanda-de Jesus Godolphim, Ricardo-Luiz-Cavalcanti Albuquerque-Júnior, Luiz-Renato Paranhos, Sigmar-de Mello Rode, Cláudio-Antonio-Talge Carvalho, Maria-Amália-Gonzaga Ribeiro

**Affiliations:** 1 DDS, MSc, Department of Restorative Dentistry, Endodontic Division, Institute of Science and Technology, São Paulo State University, São José dos Campos, SP, Brazil; 2 DDS, Department of Dentistry, Federal University of Sergipe, Aracaju, SE, Brazil; 3 DDS, MSc, PhD, Professor, Department of Dentistry, Tiradentes University, Aracaju, SE, Brazil; 4 DDS, MSc, PhD, Professor, Department of Dentistry, Federal University of Sergipe, Aracaju, SE, Brazil; 5 DDS, MSc, PhD, Professor, Department of Oral Pathology, Institute of Science and Technology, São Paulo State University, São José dos Campos, SP, Brazil; 6 DDS, MSc, PhD, Professor, Department of Restorative Dentistry, Endodontic Division, Institute of Science and Technology, São Paulo State University, São José dos Campos, SP, Brazil

## Abstract

**Background:**

Laser phototherapy (LPT) has been suggested as a new therapeutic tool to improve the repair of replanted teeth. However, its effects and mechanism of action are not yet completely understood.

**Objectives:**

This study evaluated histologically the effect of laser phototherapy (LPT) λ808 and λ660 nm on angiogenesis in the periodontal tissue of replanted teeth in rats.

**Material and Methods:**

Twenty maxillary right incisors were extracted from twenty Wistar rats and randomly assigned to four groups (n = 5): PN – teeth were stored in paper napkin for 45 min; WM – teeth were immersed in 20 ml of UHT whole cow milk for 45 min; PNL and WML – teeth received the same treatment described for PN and WM, respectively, plus LPT at λ808 and λ660 nm. All root canals were prepared and filled with calcium hydroxide paste. The animals were euthanized 15 days after tooth replantation and angiogenesis was scored by blood vessel counting in the area of periodontal ligament and alveolar bone, using the ImageJ software. Data were analyzed statistically by ANOVA and Tukey’s test (α = 5%).

**Results:**

LPT at λ808 and λ660 nm caused significant increased angiogenesis on irradiated groups (PNL and WML) when compared to the non-irradiated groups (PN and WM) (*p*<0.05). There was no statistically significant difference between PN and WM as well as between PNL and WML (*p*>0.05).

**Conclusions:**

LPT is capable of stimulating angiogenesis in vivo in the periodontal tissue of replanted teeth.

** Key words:**Angiogenesis, lasers, tooth avulsion, tooth replantation, wound healing.

## Introduction

Traumatic tooth avulsion and its treatment are challenging situations in clinical practice. Since most avulsions occur in children aged between 7 and 12 years, replantation and maintenance of the avulsed tooth in its alveolus is essential until facial growth is complete, when a permanent rehabilitative treatment may be performed (1). The traumatic injury causes disruption of the neurovascular bundle and damages multiple periodontal tissue structures, including gingival epithelium, periodontal ligament (PDL), cementum, and alveolar bone, leading to a local inflammatory response, which severity depends mainly on extra-alveolar time and storage media (2).

The immediate replantation of the avulsed tooth within 5 minutes after trauma is one of the most critical factors related to PDL healing. Delayed tooth replantation decreases the probability of periodontal healing to less than 50% (3). In this case, the tooth should be stored in a suitable media before replantation in order to maintain cell viability and minimize destructive inflammation (4). According to the American Association of Endodontics, Hank’s balanced salt solution (HBSS) is considered the best storage media for avulsed teeth, but it is not easily available at the site of accident. Thus, as delayed replantation is the most common situation in cases of avulsion, there is a continuing search for an ideal storage media with clinical efficacy equivalent to HBSS that is readily available (4,5).

Nevertheless, considering that inflammatory root resorption is the main cause of dental replantation failure even when ideal storage conditions have been used (6,7), treatment strategies that limit the extent of the periradicular inflammation, such as laser phototherapy (LPT), have been considered in recent years (8-12). The potential benefits of LPT on periodontal wound repair after tooth replantation are based on a series of biological effects such as anti-inflammatory and analgesic, which accelerate the healing process and reduce the occurrence of root resorption and ankyloses (9-13).

Angiogenesis, that is, the formation of new blood vessels from pre-existing ones, is one of the mechanisms involved in tissue repair, specifically in the proliferative phase (3–7 postsurgical days) (14). It is responsible for supplying the oxygen and nutrients necessary to sustain cell metabolism at the wound area (15,16). Moreover, the stimulation of blood vessel formation has been associated with accelerated and improved tissue repair in experimental models *in vivo* (17-19). This is why angiogenesis has been widely used as a parameter for the analysis of LPT action on the tissue repair process (14,15,20-23).

Thus, considering there are few reports on the effects of LPT on periodontal healing after tooth replantation and that its mechanism of action is not yet completely understood, this study aimed to investigate the effect of LPT (λ808 nm and λ660 nm) on angiogenesis in the periodontal tissue of replanted teeth in rats, after being stored in paper napkin or whole cow milk for 45 min. This analysis will help to elucidate aspects of the repair process in tooth replantation that so far have more often been investigated histomorphometrically (8-12).

## Material and Methods

- Experimental model and sample:

The Animal Research Ethics Committee approved this randomized single-blinded *in vivo* experimental study (process n. 041114). Twenty male Wistar rats (Rattus norvegicus albinus) weighing 200-250 g were used. The animals were housed in collective cages (49x34x16 cm) with five animals in each one, under standard conditions of temperature (22±2° C), relative humidity (55±10%), and light/dark cycle (12/12 h). They were fed with solid rations of Labina™ (Purina, São Paulo, SP, Brazil) and water ad libitum, except for the postoperative 12 h (12).

- Tooth extraction:

Before the surgical procedures, the rats were sedated and anesthetized with intraperitoneal injection of a mixture of Ketamine (Syntec do Brazil Ltda., Cotia, SP, Brazil) and Xylazine (Syntec do Brazil Ltda., Cotia, SP, Brazil) in the proportion of 0.75/0.5 ml and in a dose of 0.1 ml per 100 g of body weight. After intraoral antisepsis of the anterior maxillary portion with 0.12% chlorhexidine gluconate (Colgate-Palmolive Industrial Ltda., São Bernardo do Campo, SP, Brazil), the maxillary right incisor of each rat was extracted using a Buser elevator (Trinity Indústria e Comércio Ltda., São Paulo, SP, Brazil) and an adapted forceps #150 (Golgran Ind. Com. Instr. Odontológico Ltda., São Caetano do Sul, SP, Brazil), simulating a case of tooth avulsion (12).

- Division of groups:

Teeth were randomly assigned to four groups of 5 specimens each, according to the treatment given: PN group – teeth were stored in paper napkin (Santhe™ - Fábrica de Papel Santa Therezinha S.A., Bragança Paulista, SP, Brazil); PNL group – teeth received the same treatment described for PN plus LPT at λ808 nm and λ660 nm; WM group – teeth were immersed in 20 ml of UHT whole cow milk (Italac, Santa Helena de Goiás, GO, Brazil); WML group – teeth received the same treatment described for WM plus LPT at λ808 nm and λ660 nm. All teeth were maintained in their respective storage media for 45 minutes at room temperature (22±2° C) (12).

- Root canal treatment:

After this period, the dental papilla of each extracted tooth was excised with a #15 scalpel blade (Suzhou Kyuan Medical Apparatus Co. Ltda., Beiqiao Town, Suzhou City, China) to expose the root canal, and the pulp was extirpated via the apical foramen with a slightly pre-curved #20 Hedstrom file (Dentsply Maillefer, Ballaigues, Switzerland) in order to control the continuing growth of the rat incisor and prevent necrosis of the pulp tissue subsequent to the extraction. The root canals were gently irrigated with 5 ml of saline solution (Farmax, Divinópolis, MG, Brazil), dried with absorbent paper points (Dentsply Ind. e Com. Ltda., Petrópolis, RJ, Brazil), and filled with calcium hydroxide paste (UltraCal™ XS, Ultradent Products Inc., South Jordan, UT, USA) using 25x0.35-mm capillary tips (Ultradent Products Inc., South Jordan, UT, USA) (12).

- Tooth replantation:

Before replantation, antisepsis of the anterior maxilla with 0.12% chlorhexidine gluconate was again performed, and an alveolar lavage was done with 2 ml of saline solution for clot removal. The PNL and WML groups were treated with LPT at λ808 nm and λ660 nm according to the protocol described subsequently. Teeth were replanted into their sockets with a slow and delicate movement using clinical tweezers (Golgran Ind. Com. Instr. Odontológico Ltda., São Caetano do Sul, SP, Brazil). Soon after, the animals received a single intramuscular injection of an antibiotic (Megacilin Super Plus™, Vansil Ind. Com. e Repr. Ltda., Descalvado, SP, Brazil) diluted in 15 ml of sterile water (Samtec Biotecnologia, Ribeirão Preto, SP, Brazil), in a dose of 0.005 ml per 100 g of body weight (12).

- LPT protocol:

In the PNL and WML groups prior to replantation, the mesial and distal root surface of each tooth and the inside of the alveolus were irradiated with a gallium-aluminum-arsenate (GaAlAs) continuous-wave (CW) diode laser (Photon Lase III™, DMC Equipamentos Ltda., São Carlos, SP, Brazil), emitted at λ808 nm wavelength (infrared light), 100 mW output power, 3.6 W/cm2 power density, 1.7 J total energy per point, and 61 J/cm2 energy density. The laser was applied in a punctual mode in contact with the root surface and the alveolus entry, with a total irradiation time of 119 s, as follows: 17 s for each cervical, middle, and apical thirds of the proximal root faces (mesial/distal) and 17 s for the inside of the alveolus (12).

After replantation, another laser irradiation was carried out on the buccal and palatal mucosa of the alveolus containing the replanted tooth with an indium-gallium-aluminum-phosphorus (InGaAlP) CW diode laser (Photon Lase III™, DMC Equipamentos LTDA, São Carlos, SP, Brazil), emitted at λ660 nm wavelength (visible light) with the same parameters described for the λ808 nm GaAlAs laser, except the total exposure time was 34 s (17 s for each buccal and palatal mucosa). This procedure was repeated every 48 hours until completing five sessions of LPT at λ660 nm. The Photon Lase III™ device used in this investigation has the following characteristics: 1.9-mm laser beam diameter on the handpiece tip, 0.028-cm2 beam area, 17-degree beam divergence, and 50-degree tip angle (12).

- Euthanasia and histological processing:

The rats were euthanized by the inhalatory chemical method in carbon dioxide gas chamber, 15 days after replantation. The anatomic pieces containing the replanted teeth were removed, fixed in 10% formaldehyde for 7 days, and decalcified in 5% nitric acid for 14 days, with solution exchanges every 48 hours. After that, the pieces were dehydrated, clarified, embedded in paraffin, and sectioned (5-µm thick) in a longitudinal plane of the root (12). Three microscope slides per piece, each containing one histological section, were prepared and stained with hematoxylin and eosin (HE) for histological analysis.

**Figure 1:**
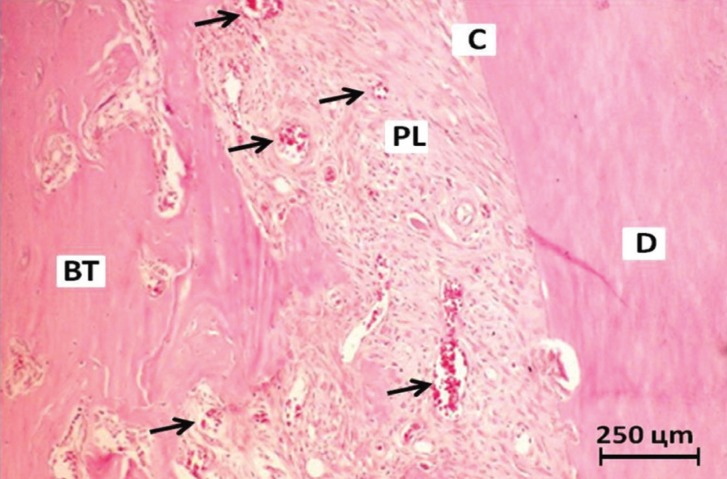
Photomicrograph of a histological section stained in hematoxylin-eosin showing an intense presence of blood vessels (arrows) on periodontal wound. Dentin (D), cementum (C), periodontal ligament (PL), bone tissue (BT). Original magnification 100×.

- Histological analysis:

Images were captured with a digital camera (Olympus Camedia C-7070; Olympus Corporation, Tokyo, Japan) attached to a microscope (Olympus CX31; Olympus Corporation, Tokyo, Japan) at 100x magnification. Histological analysis was performed only in the middle third of the palatal root face because this region is not damaged by the surgical procedures (24), and because the rat tooth has cementum and periodontal ligament only on this face and on parts of the mesial and distal faces of the root (25). Angiogenesis was measured in the area of periodontal ligament and alveolar bone by counting the number of blood vessels in two fields per histological section using ImageJ software (Wayne Rasband, National Institutes of Health, USA), without distinguishing their nature (arterioles, venules, and capillaries), as shown in Figure 1. The examiner was blinded to which groups the images belonged in order to avoid bias during the analysis.

- Statistical analysis:

The mean of blood vessels per specimen was obtained, which generated the mean per group, and consequently the standard error of the mean. These values were obtained and analyzed statistically with ANOVA and Tukey’s test after data from all groups had normality accepted by the Shapiro-Wilk test. The BioEstat 5.0 statistical software was used and the significance level was set at 5% (*p*<0.05).

Results

**Figure 2:**
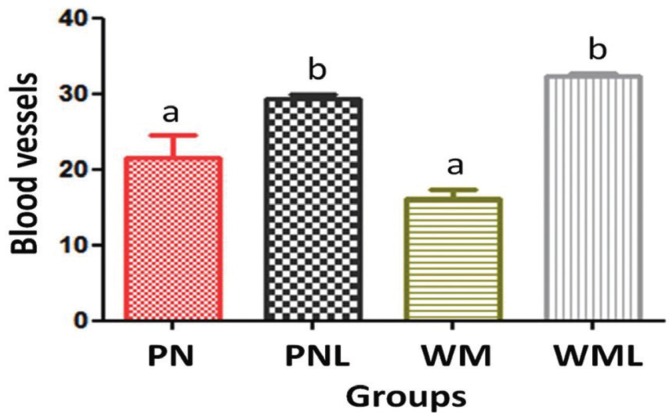
Mean ±SEM of blood vessels per group and statistical differences among the groups. Statistical analysis by ANOVA followed by Tukey’s test. Bars with different letters differ significantly among each other (*p*<0.05).

The Figure 2 shows the mean of blood vessels and standard error of the mean (SEM) per group. When comparing irradiated (PNL and WML) and non-irradiated (PN and WM) groups, statistical analysis showed a significant increase of angiogenesis in irradiated groups (*p*<0.05) (Fig. 2). Regarding the storage media for avulsed teeth used during the extra-alveolar time, there was no statistically significant difference between PN and WM (*p*>0.05) as well as between PNL and WML (*p*>0.05) (Fig. 2).

## Discussion

Several studies have been developed for decades to investigate the effects of red and/or infrared laser lights on tissue repair (8-12,14,15,20,21,23,26) but only few recent works have studied their effects on the periodontal repair process of replanted teeth (8-12). Although some reports have shown that LPT is capable of improving the periodontal repair process of replanted teeth *in vivo* by reducing the occurrence of inflammatory root resorption and ankyloses (9-12), its effects are still inconclusive because of the lack of studies, the methodological differences mainly in LPT protocols, and the many variables that may alter the response of biological tissues (12).

The present study used a combination of infrared (λ808 nm) and red laser (λ660 nm), because some studies have suggested that this association is more effective in reducing the inflammatory process and accelerating tissue repair, as different wavelengths would have different absorption and penetration (12,27-29). Irradiation of the root surface with infrared laser was based on findings of Carvalho *et al.* (9) and irradiation of the alveolar walls and vestibular/palatine mucosa with red laser was based on findings of Vilela *et al.* (10), who showed less root resorption areas in laser groups using their respective wavelengths. In order to determine whether the effect of LPT on periodontal healing after tooth replantation was positive, the specimens were microscopically assessed by means of quantitative analysis of blood vessels in the periodontal tissue, also known as angiogenesis, a key process for wound healing (14-23,30).

According to our results, 15 days after tooth replantation, LPT at λ808 nm and λ660 nm significantly increased angiogenesis in the periodontal tissue on irradiated groups (PNL and WML), indicating a positive effect of these wavelengths on periodontal healing of replanted teeth. These findings agree with previous studies that have indicated that the use of both infrared and red lasers are capable of stimulating angiogenesis *in vivo* on skin and oral wounds, resulting in a major increase of both oxygen supply and nutrients to the wound (14,20,23). Medeiros *et al.* (21) and Wagner *et al.* (15) similarly reported this phenomenon on skin and oral wound healing in rats, respectively, irradiated with red laser (λ660 nm).

The mechanism of action of LPT on angiogenesis and tissue repair can be explained through the stimulation of photosensitive chromophores such as cytochrome C oxidase, an enzyme found in the internal membrane of the mitochondria. When stimulated, these enzymes accelerate cellular metabolism throughout the increment of adenosine triphosphate (ATP) synthesis, providing energy for cellular activities that are essential for the processes of mitosis and protein synthesis (29). These events favor the healing process through increased cell proliferation, production of nucleic acids, formation of collagen, and reduction of inflammation (12,14,15,20-23,26).

Possibly, angiogenesis is also induced by laser phototherapy through different cellular signaling mechanisms. Cury *et al.* (14) showed that both infrared and red lasers increased the expression of vascular endothelial growth factor (VEGF), hypoxia inducible factor (HIF-1α), and modulated matrix metalloproteinase (MMP-2) activity, which are three major mediators involved in angiogenesis. The VEGF plays a key role in angiogenesis by stimulating the proliferation and migration of endothelial cells and HIF-1α regulates the cellular response to hypoxia by activating genes that are important to cellular adaptation under hypoxic conditions. The activity of MMP-2 is necessary for vascular basement membrane degradation and extracellular matrix remodeling, in order to allow the migration of endothelial cells during angiogenesis (14).

Cytokine modulation is another basic mechanism whereby LPT increases angiogenesis and improves tissue repair (16). Recent experimental research has showed that LPT at λ660 nm increased the tissue levels of IL-1β at the early stage of oral wound healing and decreased the tissue levels of TNF-α during all stages of oral wound healing (15). Among its several roles during wound healing, IL-1β is capable of acting directly and indirectly on endothelial cells by activating infiltrating cells to produce endothelial cell-activating factors such as VEGF. Thus, the increase in IL-1β tissue levels induced by LPT at the early (inflammatory) phase of wound healing could be associated with its pro-angiogenic effect. The TNF-α is a pro-inflammatory cytokine that, in excess, is associated with chronic and slow-healing acute wounds (15,30).

A greater proliferation of blood vessels was expected when whole cow milk was used as storage media, because it presents better conditions for the periodontal repair process by providing some nutrients and growth factors (12,31,32). Paper napkin, on the other hand, does not provide any benefit to maintaining the vitality of PDL cells, besides presenting higher risk of bacterial contamination (12). However, the results of this research revealed that the effects of storage solutions on angiogenesis were similar for both whole cow milk and paper napkin, which can be justified by the triggering of a local adaptive response through a physiological mechanism of compensation to the nutritional deficiency caused by the dry environment.

In a previous study (12), our team showed, through histomorphometric analysis, that whole cow milk, as well as soy milk, provided better conditions for the periodontal repair process, reducing the occurrence of root resorption and ankylosis. This finding suggests that the storage media can improve the repair process by other biological mechanisms involved in periodontal repair, such as favoring the regeneration of the PDL by providing essential metabolites for cell maintenance (12,33-35), and not stimulating angiogenesis. Regarding the effects of LPT, the findings of the present investigation are in agreement with previous studies that observed a significant improvement in the process of periodontal repair in the irradiated groups (9-12), confirming the assumption that the stimulation of blood vessel formation is associated with accelerated and better tissue repair (17-19).

We may conclude that LPT at λ808 nm and λ660 nm improved the periodontal repair 15 days after tooth replantation as evidenced by the increased blood vessel formation or angiogenesis, regardless of the storage media. Thus, LPT could be used as a new therapeutic strategy to accelerate and improve the periodontal repair process of replanted teeth. Nevertheless, further studies should be performed to clarify red and infrared laser effects on periodontal healing and to confirm its correlation with angiogenesis.
